# Vitamin D Receptor Gene Polymorphism and Left Ventricular Hypertrophy in Chronic Kidney Disease

**DOI:** 10.3390/nu6031029

**Published:** 2014-03-10

**Authors:** Domenico Santoro, Giorgia Gagliostro, Angela Alibrandi, Riccardo Ientile, Guido Bellinghieri, Vincenzo Savica, Michele Buemi, Daniela Caccamo

**Affiliations:** 1 Department of Clinical and Experimental Medicine, University of Messina, Messina 98123, Italy; E-Mails: giorgiagagliostro@tiscali.it (G.G.); gbellinghieri@unime.it (G.B.); visavica@tin.it (V.S.); buemim@unime.it (M.B.); 2 Department of Economical, Business and Environmental Sciences and Quantitative Methods, University of Messina, Messina 98123, Italy; E-Mail: aalibrandi@unime.it; 3 Department of Biochemical, Physiological and Nutritional Sciences, University of Messina, Messina 98123, Italy; E-Mails: riccardo.ientile@unime.it (R.I.); dcaccamo@unime.it (D.C.)

**Keywords:** vitamin D receptor, gene polymorphisms, left ventricular hypertrophy, parathormone, chronic kidney disease

## Abstract

FokI and BsmI polymorphisms of vitamin D receptor (VDR) gene are regarded as reliable markers of disturbed vitamin D signaling pathway. Left ventricular hypertrophy (LVH) is a strong cardiovascular risk marker in end stage renal disease (ESRD) patients. Since BsmI polymorphism has been associated with LVH in ESRD patients, we addressed this study in patients with chronic kidney disease (CKD) not yet on dialysis. One hundred and forty five patients with CKD stage 3 were genotyped for FokI and BsmI VDR polymorphisms, in order to assess the relationships between these VDR polymorphisms, some markers of mineral bone disorders, and LVH measured by echocardiography. Patients bearing either the Ff heterozygous or FF homozygous genotype had significantly higher PTH values than those bearing the ff genotype. The relationships between VDR genotypes and LVH revealed a highly significant association of the BsmI Bb heterozygous genotype with LVH. In patients with CKD stage 3 BsmI B allele was independently related to LVH. Since LVH is a frequent finding in dialysis population due to several mechanisms, the presence of the same relationship in patients with CKD strengthens the hypothesis that alterations of vitamin D signaling are implicated in LVH development in patients with renal diseases.

## 1. Introduction

Several studies indicate a relationship between vitamin D, survival, vascular calcification and inflammation [1,2,3]. In addition to its central role in the regulation of bone mineral metabolism, vitamin D is also involved in the regulation of other systems, including the immune, cardiovascular and endocrine. The biological activity of vitamin D is mediated by the activation of the high-affinity nuclear vitamin D receptor (VDR) [4]. VDR is a ligand-dependent transcription factor, belonging to the steroid nuclear receptor gene family, that, after activation, heterodimerizes with retinoid X receptor and binds to specific DNA sites to modify the expression of target genes [5,6]. Most, if not all, pleiotropic actions of vitamin D and its analogues are specifically mediated by VDR. Left ventricular hypertrophy (LVH) is a strong cardiovascular risk marker in end stage renal disease (ESRD) patients [7]. Due to the relevant role of vitamin D in heart disease, the association between LVH and VDR gene polymorphisms has been recently investigated. Indeed, it has been reported that the VDR BsmI gene polymorphism is involved in LVH in ESRD patients [8,9]. In particular, BsmI polymorphism resulted independently related to LVH and LVH progression in dialysis patients, and the frequency of the BsmI B mutated allele as well as the number of B alleles was directly related to left ventricle mass index (LVMI) in this population [8]. For this reason, the presence of BsmI mutated variant of VDR gene in ESRD patients has been proposed as a novel marker of disturbed vitamin D signaling pathway, determining consequently an increase in left ventricle mass (LVM) [9]. Since changes in LVM are very frequent in hemodialysis (HD) patients and may be related to both pressure overload and several hemodynamic as well as non-hemodynamic factors [10], we aimed to evaluate the effects of alterations in VDR signaling in a population of patients with chronic kidney disease (CKD) not yet on dialysis. Therefore, we carried out a genotyping approach investigating the distribution of FokI and BsmI VDR gene polymorphisms in a cohort of patients with CKD stage 3 in order to assess the relationships between different VDR alleles and genotypes and LVM in this population.

## 2. Experimental Section

### 2.1. Subject Recruitment

The patients included in this study were a Sicilian cohort of subjects (63 F, 82 M; age 66 ± 16.4 years) affected by CKD stage 3b who were referred to the Center for Nephrology and Dialysis at the University Hospital of Messina (Italy), for clinical evaluation. Moreover, in order to carry out a comparison between the genetic background of the recruited patients and that of the general population from the same geographic area we also recruited 130 healthy subjects (75 M/55 F; 47.1 ± 10.2 years). Peripheral blood samples were drawn from all subjects after release of informed consent. The study design is consistent with the principles of the Declaration of Helsinki.

### 2.2. Biochemical Assays and Echocardiography

CKD patients were classified according to GFR, using CKD-EPI equation. Since all patients were evaluated in our hospital, serum creatinine in this cohort was measured using an enzymatic technique (modular P Analyzer, creatinine plus assay; Roche Diagnostics, Mannheim, Germany). Urinary creatinine measurement, as well as determination of plasma concentrations of calcium, phosphate, vitamin D and parathormone (PTH), were made using routine laboratory methods. Echocardiographic measurements were carried out in the same day of blood sampling, according to the recommendations of the American Society of Echocardiography by an observer unaware of biochemical results. LVM was calculated according to the Devereux formula and indexed to height^2.7^ [10]. LVH was defined by an LVMI of over 47 g/m^2.7^ in women or over 50 g/m^2.7^ in men.

### 2.3. Genotyping for VDR Polymorphisms

Genomic DNA was extracted from peripheral blood lymphocytes by Gentra Puregene Blood kit (Qiagen, Milan, Italy). Then, genotyping for FokI and BsmI VDR polymorphisms was carried out by Real-time PCR using TaqMan Genotyping Assays (C_12060045_20; C_8716062_10) (Applied Biosystems, Monza, Italy).

### 2.4. Statistical Analysis

The between-groups variability was analysed by Mann-Whitney test. A Logistic Regression Model for LVH was estimated in order to evaluate the dependence of some variables, such as age, gender, diabetes, uncontrolled hypertension (patients unresponsive to antihypertensive treatment) and FokI or BsmI genotypes [11]. Statistical analyses were performed using SPSS 11.0 for Windows package.

## 3. Results

Clinical and biochemical features of recruited CKD 3b patients are shown in Table 1. Only one fifth of patients had no co-morbidities, while more than one third were also suffering from LVH, and 25% were also suffering from hypertension and LVH. Moreover, 90% of subjects with diabetes were also suffering from both hypertension and LVH. Genotyping for VDR polymorphisms showed that the distribution of VDR FokI and BsmI genotypes in CKD 3b patients was not significantly different (*p* = 0.32 and *p* = 0.18, respectively) from the one observed in the healthy general population of the same geographic area (Table 2). Genotype frequencies were in Hardy-Weinberg equilibrium both in CKD patients (FokI: chi-square = 1.52, *p* = 0.21; BsmI: chi-square = 3.20, *p* = 0.07) and healthy subjects (chi-square = 0.61, *p* = 0.43 for FokI; chi-square = 3.20, *p* = 0.07 for BsmI). The homozygous mutated FF genotype as well as the heterozygous Bb genotype were the most frequent among CKD 3b patients (Table 2). The comparison of allele frequencies showed that the b wild-type and the F mutated alleles, known to be associated, respectively, with higher VDR protein amounts and transcriptional activity than the ones observed in the presence of B and f alleles [12], were significantly more frequent than B mutated and f wild-type alleles. Notably, the F allele was more represented in the homozygous than in the heterozygous state, while the B allele was mostly represented by heterozygous individuals (Table 2). The analysis of between-groups variability of biochemical parameters showed that patients bearing either the Ff heterozygous or FF homozygous genotype had significantly higher PTH values than those with ff genotype. Notably, patients with BsmI BB genotype had PTH values significantly higher than those bearing other BsmI genotypes (Table 3). We also investigated the association between either VDR FokI or BsmI polymorphim with co-morbidities observed in CKD patients, *i.e.*, diabetes, hypertension, and LVH. Interestingly, we found a strongly significant association of the BsmI B allele with LVH, given the highest percentage of LVH occurrence in patient subgroups carrying either the Bb or BB genotype, while no association was found for the BsmI b allele or FokI variants with different co-morbidities (Table 3). The logistic regression model for LVH showed that BsmI B allele is associated with LVH independently from other common causes of ventricular hypertrophy (Table 4). Moreover, the odds ratio calculation showed that the risk for LVH was increased by 21.3 fold (95% CI = 8.4–55.01, *p* < 0.0001) in subjects bearing the BsmI Bb heterozygous genotype and by 40 fold (95% CI = 7.8–204.2, *p* < 0.0001) in patients with the BB homozygous genotype. This suggests that the presence of two BsmI B mutated alleles has an additive effect in terms of molecular pathogenesis of LVH in CKD 3b patients. There were no statistical differences in 25-OH-Vitamin D levels between either VDR FokI or BsmI different genotypes, even when considering median values. However, vitamin D deficiency (<15 ng/ml) was prevalent in the FF group and Bb group, showing a frequency (37%) higher than in other groups (BB = 25%; ff = 17%; Ff = 14%; bb = 11%).

**Table 1:** Biochemical and Clinical data of the sampled chronic kidney disease (CKD) 3b population.

Biochemical data	Median values ± DS	Reference range
Calcium (mg/dL)	8.7 ± 0.6	9–10.7
Phosphate (mg/dL)	3.6 ± 0.8	2.4–4.1
25-OH-Vitamin D (ng/mL)	24.3 ± 17.1	40–120
Parathormone (pg/mL)	32.7 ± 19.1	11–54
Comorbid conditions	Number of patients	
None	31 (21.4%)	-
LVH	49 (33.9%)	-
Uncontrolled Hypertension	11 (7.6%)	-
Uncontrolled Hypertension/LVH	35 (24.1%)	-
Diabetes mellitus/Uncontrolled Hypertension	5 (3.4%)	-
Diabetes mellitus/LVH/Uncontrolled Hypertension	14 (9.6%)	-

*Note:* Uncontrolled Hypertension was defined as Blood Pressure > 140/90 mmHg despite anti-hypertensive treatment.

**Table 2:** Vitamin D receptor (VDR) genetic background of the sampled CKD 3b population.

Genotype	Number of patients	Healthy subjects
FokI f/f (TT)	14 (9.7%)	11 (8.5%)
FokI F/f (TC)	52 (35.8%)	45 (34.6%)
FokI F/F (CC)	79 (54.5%)	74 (56.9%)
Allele F frequency	0.72	0.74
Allele f frequency	0.28	0.26
BsmI b/b (GG)	49 (33.8%)	47 (36.1%)
BsmI B/b (AG)	76 (52.4%)	69 (53.1%)
BsmI B/B (AA)	20 (13.8%)	14 (10.8%)
Allele B frequency	0.40	0.37
Allele b frequency	0.60	0.63

**Table 3:** Variability of biochemical markers and distribution of co-morbid conditions in relationship to FokI and BsmI VDR genotypes in CKD 3b patients.

	*ff (n=14)*	*Ff (n=52)*	*FF (n=79)*	*bb (n=49)*	*Bb (n=76)*	*BB (n=20)*
Calcium (mg/dL)	8.8 ± 0.4	8.3 ± 1.9	8.7 ± 0.6	8.7 ± 0.5	8.8 ± 0.5	8.6 ± 0.4
Phosphate (mg/dL)	3.4 ± 0.7	3.4 ± 0.5	3.5 ± 0.6 ^#^	3.5 ± 0.4	3.4 ± 0.6	3.3 ± 0.3
25-OH-Vitamin D (ng/mL)	28.8 ± 23.7	26.1 ± 21.1	22.0 ± 13.1	25.9 ± 9.5	24.2 ± 17.7	24.8 ± 16.5
Parathormone (pg/mL)	25.5 ± 16.4	38.5 ± 17.4 *	33.4 ± 12.6 *	36.4 ± 17.7	31.9 ± 24.4	45.3 ± 9.5 ^¥^
Diabetes	2 (14.3%)	9 (17.3%)	8 (10.1%)	4 (9.1%)	15 (18.5%)	0 ^§§§^
Uncontrolled Hypertension	9 (64.3%)	26 (50.0%)	31 (39.2%)	18 (40.9%)	46 (56.8%)	1 (5%) ^§§§^
LVH	9 (64.3%)	37 (71.1%)	52 (65.8%)	9 (18.4%)	63 (82.9%) ^§§§^	18 (90%) ^§§§^

*Note:* * *p* < 0.05, ** *p* < 0.01, significant values in comparison with wild-type ff genotype. ^#^ *p* < 0.05, significant value in comparison with heterozygous Ff genotype. ^¥^ *p* < 0.05, significant value in comparison with other BsmI genotypes; ^§§§^ *p* < 0.001 significant value in comparison with all other genotypes.

**Table 4:** Results of logistic regression model for left ventricular hypertrophy (LVH) in CKD 3b patients.

Covariates	B coefficient	*p*	OR
Constant	−3.947	0.057	0.019
B allele	4.140	0.000*	62.794
FokI	0.470	0.340	1.599
Age	0.007	0.749	1.007
Gender	0.482	0.482	1.620
Diabetes mellitus	−1.180	0.197	0.307
Uncontrolled Hypertension	0.825	0.335	2.281

*Note:* * *p* < 0.05 significant value in comparison to other covariates.

## 4. Discussion

Our study shows that the VDR BsmI B mutated allele is independently related to LVH in a population of CKD patients not on dialysis, and may be considered as a risk genetic factor for the development of LVH at stage 3b of CKD. Indeed, we found that the presence of the BsmI B mutated allele either in heterozygous or homozygous state conferred a disease risk dramatically higher (21–40 fold) than the presence of the b wild type allele. Similarly, other authors [8,9] found that the VDR BsmI B gene variant is independently related to LVH in patients on dialysis, and displays an allele dose-dependent effect on the development of LVH. In any case, these patients are subject to pressure overload but also to several hemodynamic and non-hemodynamic factors and for this reason cannot be completely assimilated to patients with CKD stage 3b. Pleiotropic effects of VDR activation are strongly affected by the presence of different VDR gene polymorphisms [4]. Indeed, most studies have been aimed at determining whether VDR polymorphisms could be involved in the development of secondary hyperparathyroidism (sHPT), one of the main complications in patients with CKD. Interestingly, the VDR BsmI b allele was shown to have a higher incidence in HD patients with sHPT and drive the progression toward sHPT in patients with ESRD, since BB individuals had lower levels of PTH and higher calcitriol levels than bb individuals in every stage of CKD [13,14,15,16,17]. Serum PTH levels in CKD patients were also affected by FokI polymorphism, since patients in the FF group had significantly higher PTH levels than those in both the Ff and ff groups, while no significant differences in serum levels of calcidiol or calcium were found among genotypes [18]. Our results confirm previously reported observations since we found that CKD 3b patients having either the bb, FF or Ff genotype had significantly higher PTH levels than patients bearing FokI ff or other BsmI genotypes. Notably, the VDR BsmI polymorphism, together with age, diabetes and calcitriol treatment, has been shown to strongly influence survival in hemodialysis patients, whereas the poly-A and FokI polymorphisms have not [19]. In particular, a longer life expectancy was observed in hemodialysis patients having the bb genotype than in those having either the Bb or BB genotype [19]. There are two possible mechanisms through which the BsmI polymorphism could influence mortality: (1) by directly modifying the VDR sensitivity or expression in target organs, particularly cardiac and vascular tissues or 2) by modifying the circulating levels of vitamin D levels [19]. In this regard, a direct influence of the BsmI B allele on cardiac tissue was shown since a higher incidence of LVH was observed in patients having a BsmI mutated genotype [8]. More recently, some authors found a positive correlation between the number of B alleles and LVMI, and significantly lower serum concentrations of 25-hydroxyvitamin D in patients with BB genotype compared to both Bb and bb genotypes [9]. Our results did not agree with these observations since in our patients 25-hydroxyvitamin D concentrations were lower in bb patients group, although this difference was not found to be significant, likely due to the small size of the sampled CKD 3b population. Moreover, in the PRIMO trial, which included 227 patients with CKD stage 3-4 and LVH by echocardiography randomized to paricalcitol or placebo, the authors showed that changes in LVM index by MRI after 12 months were not different between the two groups. Anyway, the above-specified end point of cardiovascular-related hospitalizations was reduced in the paricalcitol-treated group (*P* = 0.04), thus demonstrating a correlation between VDR activation and reduced cardiovascular events [20]. Recently, similar results were reported in the OPERA trial. In this study, patients with 3–5 CKD were randomly assigned to receive oral paricalcitol or matching placebo. After 52 weeks, VDR activation with paracalcitol significantly improved sHPT, but failed to demonstrate any change in the measurement of left ventricle structure and function in patients with severe CKD [21]. The use of VDR polymorphisms as diagnostic tools is still debated because the association of a certain VDR polymorphism with a phenotype does not necessarily imply a causative role of the polymorphism. However, genotyping for VDR variants might help to improve the management of renal diseases given that VDR genotypes have been shown to influence the response to therapy. For example, the BsmI genotype has also been shown to affect the response of HD patients to a single bolus of calcitriol, since a higher reduction of PTH levels could be observed in patients having the BB genotype than in patients with the bb genotype, even after correcting by calcium and phosphorus levels [22]. In particular, it could be intriguing to investigate whether long term treatment with vitamin D may reverse or modify this relationship between BsmI B variant and LVH.

## 5. Conclusions

In this study we demonstrated for the first time that BsmI B allele is independently related to LVH in patients with CKD stage 3b. The small size of sampled population included in this genotyping study represents a limitation and did not allow us to draw definitive conclusions on the role of VDR genotypes and cardiovascular complications. However, as already shown for ESRD patients [8], it is possible to add another case to the previously postulated theory that altered vitamin D signaling, even in patients with CKD stage 3b, is implicated in LVH development.
